# BirdNeRF: fast neural reconstruction of large-scale scenes from aerial imagery

**DOI:** 10.1038/s41598-025-21206-z

**Published:** 2025-10-24

**Authors:** Huiqing Zhang, Yifei Xue, Ming Liao, Yizhen Lao

**Affiliations:** 1 College of Computer Science and Electronic Engineering, Hunan University, Changsha, 410082 China; 2 College of Information and Computer Engineering, Pingxiang University, Pingxiang, 337055 China; 3 School of Design, Hunan University, Changsha, 410082 China; 4 Jiangxi Provincial Natural Resources Cause Development Center, Nanchang, 330002 China

**Keywords:** NeRF, Large-scale reconstruction, Aerial image, Spatial decomposition, Projection-guided, Computer science, Information technology

## Abstract

In this study, we introduce BirdNeRF, an adaptation of Neural Radiance Fields (NeRF) specifically designed for reconstructing large-scale scenes using aerial imagery. Unlike previous research which focused on small-scale and object-centric NeRF reconstruction, our approach addresses multiple challenges, including (1) Addressing the issue of slow training and rendering associated with large models. (2) Meeting the computational demands necessitated by modeling a substantial number of images, requiring extensive resources such as high-performance GPUs. (3) Overcoming significant artifacts and low visual fidelity commonly observed in large-scale reconstruction tasks due to limited model capacity. Specifically, we present a novel bird-view pose-based spatial decomposition algorithm. This algorithm decomposes a large aerial image set into multiple small sets with appropriately sized overlaps, allowing us to train individual NeRFs of sub-scene. This decomposition approach enables rendering to scale seamlessly to arbitrarily large environments. Moreover, it allows for per-block updates of the environment, enhancing the flexibility and adaptability of the reconstruction process. Additionally, we propose a projection-guided novel view re-rendering strategy, which aids in effectively utilizing the independently trained sub-scenes to generate superior rendering results. We evaluate our approach on existing datasets as well as against our own drone footage, achieving a reconstruction speed improvement of 10x over classical photogrammetry software and 50x over the state-of-the-art large-scale NeRF solution, all on a single GPU with comparable or superior rendering quality.

## Introduction

Large-scale 3D reconstruction at a city-wide level is an intrinsically active and significant task in the fields of photogrammetry and remote sensing. This process focuses on creating detailed and accurate 3D models of entire cities using a variety of data sources, including, but not limited to, aerial or satellite images, LiDAR data, and street-level imagery. The rapid advancements in aerial surveying technology have made the acquisition of high-resolution images simpler and more cost-effective. As a result, image-based 3D reconstruction has emerged as a rich and promising area of study, encompassing numerous applications.

3D urban models find extensive application across various fields, significantly enhancing them. For instance, urban development benefits from these models as they enable simulations and visual demonstrations of various scenarios, such as the construction of new buildings, the implications of transportation projects, and the layout of public spaces, thereby supporting informed decision-making^1^. In the sphere of navigation, 3D urban reconstructions contribute to the creation of accurate and exhaustive maps to improve the precision of GPS devices and mobile applications^2^. Furthermore, this technology serves as the foundation for augmented reality applications, providing real-time overlaid directions on real-world views. Virtual tourism flourishes with extensive 3D reconstructions, allowing individuals to explore cities virtually before visiting^3^. For the real estate industry, such reconstructions offer potential buyers a clear and intuitive understanding of a property’s surroundings, facilitating property valuation^4^. 3D reconstruction also hastens rescue operations, damage assessments, and post-disaster reconstruction planning during disaster management^5^. The domain of historical preservation considerably benefits from 3D reconstructions by aiding research, cultural heritage preservation, and the creation of virtual reconstructions for lost or damaged historical sites^6^. These diverse applications highlight the significance of 3D city modeling across various sectors.

Existing image-based 3D reconstruction techniques can be divided into two broad categories: traditional geometry-based methods and neural network-based methods. Geometry-based methods involve a two-step process primarily consisting of Structure-from-Motion (SfM) and Multi-View Stereo^7^(MVS). SfM estimates camera poses and sparse 3D points from input images^8^, while MVS refines point clouds and constructs a dense 3D model^9^. Neural network-based methods, exemplified by Neural Radiance Fields^10^(NeRF) , represent a groundbreaking advancement in 3D reconstruction. NeRF utilizes neural networks to implicitly represent^11,12^ three-dimensional scene data by training network parameters based on input images and their corresponding camera poses. It also depicts the ability to generate novel viewpoint images. Recent advancements in NeRF research, such as Instant Neural Graphics Primitives^13^(Instant-NGP), highlight the rapidly evolving landscape of this field. However, current large-scale urban reconstruction methods face three main challenges:

1. **Slow Training and Rendering with Large Models:** Large-scale reconstruction processes are inherently time-consuming. As the demand for real-time or near real-time applications, such as navigation and disaster management, intensifies, research is needed into faster and more efficient large-scale 3D reconstruction techniques.
2. **Computational Demands:** Large-scale reconstruction involves handling and processing vast datasets, often exceeding the memory capacity of a single GPU. This can lead to slow processing times, out-of-memory errors, and other performance-related issues, posing challenges for users and researchers with limited memory resources.
3. **Artifacts and Low Visual Fidelity:** Traditional geometry-based 3D reconstruction methodologies often face challenges related to inaccurate camera pose estimation and limitations in model capacity. These issues manifest as artifacts and gaps in the reconstruction, leading to suboptimal visual fidelity.

**Fig. 1:**
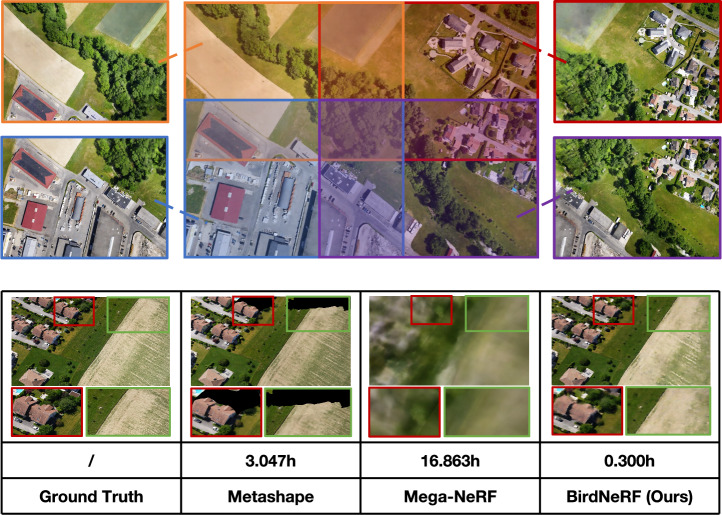
Illustration of modular scene training, along with performance and time comparisons on the IZAA dataset, which includes 1469 images. We demonstrate an approximately 10x speed improvement over traditional Metashape software. Moreover, when compared to current large-scale reconstruction approaches utilizing deep learning, our method exhibits an approximately 56x enhancement in speed.

Driven by the complex challenges of large-scale scene reconstruction from aerial imagery, especially when using NeRF, we propose BirdNeRF in this study. BirdNeRF is a specialized adaptation of NeRF crafted for reconstructing large-scale scenes. This work is inspired by the classic divide-and-conquer algorithm. BirdNeRF chiefly incorporates spatial decomposition of camera distribution, followed by the modular training of smaller scenes (Fig. 1), and finally generates images from novel viewpoints through our distinctive projection-guided view re-rendering strategy. BirdNeRF not only enables reconstruction based on extensive aerial survey image inputs but also ensures high standards of reconstruction quality and modeling speed. Figure 1 illustrates a comparative analysis of reconstruction quality between our proposed BirdNeRF and several other large-scale reconstruction methods. It also presents a comparison of training durations. Both analyses are based on a dataset comprising 1469 images. The results depict significant improvements over previous solutions, proof of the efficacy of BirdNeRF in effectively addressing the challenges identified.

## Related work and motivations

### Related work

#### Structure-from-motion and multi-view-stereo

Structure-from-Motion (SfM)^14^ and Multi-View Stereo (MVS)^9^ together create a robust pipeline for 3D reconstruction^7^. SfM focuses on recovering camera poses and a sparse 3D structure of the scene. MVS aims to generate a dense 3D representation by estimating the depth or disparity of each pixel in the images, resulting in a dense point cloud. Frequently, MVS extends beyond mere dense reconstruction and integrates surface reconstruction methods^15,16^, resulting in either a mesh or continuous surface representation derived from the dense point cloud.

Prominent SfM and MVS techniques such as VisualSfM^17^, COLMAP^8^, and OpenMVG^18^ have made significant advancements. However, these methods can be hindered by scalability issues, slow processing speeds, and visual deficiencies, including holes, texture blending, and distortion^19^. Insufficient information and inadequate image coverage in certain regions can lead to the problem of holes. Errors in camera calibration, image noise, and inaccurate feature matching can cause texture blending and visual distortions, among other issues. Various post-processing techniques, such as hole filling, texture blending corrections and mesh refinement, are required to mitigate these challenges^20^. Consequently, there is a concerted effort in ongoing research to improve the accuracy, efficiency, and scalability of SfM and MVS algorithms, particularly for large-scale reconstruction tasks.

#### Neural radiance fields

NeRF^10^ utilizes a deep neural network to model the volumetric scene as a continuous function, eliminating the necessity for explicit geometrical or point-based representation. NeRF learns from a scene to predict the appearance and density at any given 3D point, generating high-quality 3D reconstructions and novel views. NeRF involves significant computational expenses, especially during training and rendering. Tackling this issue is essential for progressing current technology.

Several extensions of naive NeRF have been developed to enhance this method. For instance, Instant-NGP^13^ employs a hash encoding to accelerate the process remarkably, making it the fastest NeRF method available today. Similarly, NeRF++^21^ and Mip-NeRF 360^22^ have been specifically designed for unbounded scenes. DeRF^23^ divides the scene using spatial Voronoi and renders each image part independently, achieving a rendering speed three times faster than NeRF^5^. KiloNeRF^24^ segments the scene and allocates it to thousands of small networks for collective training, which speeds up the inference process to some degree. However, both DeRF and KiloNeRF require an additional costly initialization^5^. PixelNeRF^25^ optimizes the NeRF model training by leveraging prior information from image features, facilitating rapid model reconstruction from sparse inputs. Nevertheless, none of these methods is suitable for city-level reconstruction.

#### Object detection and tracking methods

BEVFormer^26^ introduces a spatio-temporal transformer framework that generates bird’s eye view representations from multi-camera inputs, significantly advancing 3D perception tasks such as object detection and map segmentation in autonomous driving. MonoCT^27^ introduces a domain adaptation framework for monocular 3D object detection that tackles cross-viewpoint variations among automotive, traffic camera, and drone perspectives by leveraging generalized depth enhancement and consistency-driven pseudo-label refinement. Zheng et al.^28^ propose a BoxCloud representation that encodes point-to-boundary box distances to enhance feature discrimination for single-object 3D tracking in sparse and occluded LiDAR point clouds. Zheng et al.^29^ propose M2-Track, a motion-centric two-stage 3D single-object tracking paradigm that predicts inter-frame target motion and refines bounding boxes via motion-assisted shape completion, achieving enhanced robustness and accuracy over appearance-based methods in sparse LiDAR point clouds. DMT^30^ introduces a lightweight motion-based 3D single-object tracking framework that predicts target centers via temporal motion modeling and refines bounding boxes through an explicit voting mechanism, achieving real-time performance and improved accuracy without relying on complex 3D detectors. Wu et al.^31^ propose a target-aware projection module and IoU-guided matching distillation to effectively transfer 2D tracking knowledge to 3D point cloud tracking, significantly improving accuracy and real-time performance in sparse 3D data scenarios.

#### Commercial reconstruction software

Pix4D Mapper^32^ and Agisoft Metashape^33^ are both well-known and widely used commercial software packages that offer comprehensive solutions for photogrammetry and 3D reconstruction. They provide robust and reliable ways to process aerial and terrestrial images, generating accurate and detailed 3D models, point clouds, orthomosaics, and digital surface models. Benefiting from well-established algorithms for camera calibration, feature matching, dense point cloud generation, and mesh reconstruction, they deliver exceptional reconstruction quality.

Despite their capabilities, these software tools possess inherent limitations and challenges. Photogrammetry and 3D reconstruction processes are computationally intensive, particularly when dealing with large datasets or highly complex scenes. Consequently, both Pix4D Mapper and Agisoft Metashape require substantial computational resources, including processing power, memory, and ample storage. Users attempting to run these softwares on lower-end machines or systems with limited resources may experience sluggish processing times and diminished performance.

#### Large scale reconstruction

Over the past few decades, extensive efforts have been made towards achieving large-scale reconstruction. Studies such as Snavely et al.^34^ and Agarwal et al.^35^ focus on employing parallelism in the reconstruction process. A significant breakthrough in large-scale reconstruction tasks has been achieved by these works^36–38^ through the application of SfM and MVS methods. For instance,VLSG-SfM^36^ introduces a divide-and-conquer framework for handling large-scale global SfM, while DVLS-BA^37^ proposes a distributed method to address global bundle adjustment for large-scale SfM computations. LS-MVS^38^ employs surface-segmentation-based camera clustering to achieve the decomposition of large-scale MVS. These novel approaches provide significant inspiration for our work.

Several examples^5,19,39^ of large-scale reconstruction works are based on NeRF. Despite Mega-NeRF^5^ achieving large-scale reconstruction on a single GPU, it suffers from extremely long training times due to its reliance on the original NeRF implementation. Block-NeRF^19^ primarily uses images captured by vehicle-mounted cameras and decomposes the scene into distinct spatial units, each corresponding to a fixed city block. However, this method requires significant training resources. Furthermore, BungeeNeRF^39^ models diverse multi-scale scenes using multiple data sources. Although these methods have successfully achieved large-scale reconstruction, they have done so at the cost of extensive training resources, while also failing to effectively addressing the challenge of limited GPU memory in practical applications.

### Motivations and contributions

From the above discussion, it is clear that a fast, high-quality, large-scale 3D reconstruction methodology that operates within limited resource constraints has yet to be developed. The existing methods, especially those based on NeRF for 3D modeling, impose considerable demands on the training resources. This high computational burden, along with extended training durations, often results in limited model capacity, leading to visual distortions such as artifacts, voids, and blurring. A quick and high-quality modeling process is imperative, especially in critical fields such as urban planning and disaster response. Therefore, exploring expedited, high-quality, large-scale 3D reconstruction methods that work within limited memory constraints is particularly important and essential. These efforts hold significant potential for catering to the growing and changing needs of such crucial applications.

To address the significant challenge of achieving fast, high-quality, large-scale 3D reconstruction under limited memory constraints, we introduce BirdNeRF. Our approach begins with spatial decomposition, leveraging the spatial distribution of cameras to identify distinct clusters and segment the training scenes accordingly. Each resulting sub-scene is then trained independently. In the final stage, we employ a novel projection-guided novel view re-rendering strategy to register and align the queried camera with the corresponding sub-scenes, enabling efficient rendering of the requested viewpoints. This systematic approach, combining spatial decomposition, independent training, and a customized re-rendering strategy, optimizes large-scale 3D reconstruction in resource-constrained environments. We conducted extensive experiments to assess our method, offering both qualitative and quantitative evaluations of its effectiveness. The results demonstrate clear advantages in modeling time and rendered image quality. The main contributions of this work are summarized as follows:

1. **Fastest large-scale reconstruction:** We present a pipeline for large-scale reconstruction that achieves the fastest speed to date. Our method can reconstruct scenes covering up to 1 square kilometer in roughly 30 minutes, surpassing commercial software like Metashape by a factor of ten or more, and outperforming current deep learning approaches by over fifty times. This speed advantage becomes even more pronounced with larger datasets.
2. **Adaptability to GPU memory constraints:** Our methodology is distinguished by its flexibility in accommodating various GPU memory resources. By employing a spatial decomposition strategy based on camera distribution, we effectively manage large-scale reconstructions within limited GPU memory. This adaptability ensures our method remains versatile and scalable, demonstrating its applicability across diverse hardware configurations.
3. **Innovative re-rendering strategy for high-quality results:** We present a novel projection-guided novel view re-rendering strategy that ensures precise camera registration and querying throughout the rendering process. This strategy carefully incorporates relevant sub-models to produce the final output, ensuring accurate and efficient integration of rendered images from specific viewpoints across various sub-scenes.

## Methodology

Representing and reconstructing large-scale scenes, such as those found in aerial imagery, presents significant challenges due to the inherent scalability limitations of training a single NeRF. To address these issues, we propose BirdNeRF, a method characterized by decomposing the environment into a series of NeRFs, each trained individually based on the bird’s-eye view field of view (FOV). During the inference phase, the novel view is rendered by aggregating the outputs from these disparate NeRFs. This strategy, which we term the“split-unite paradigm”, successfully circumvents the limitations of model capacity that have stymied previous NeRF research, enabling efficient reconstruction of expansive scenes even with limited computational resources.

**Fig. 2:**
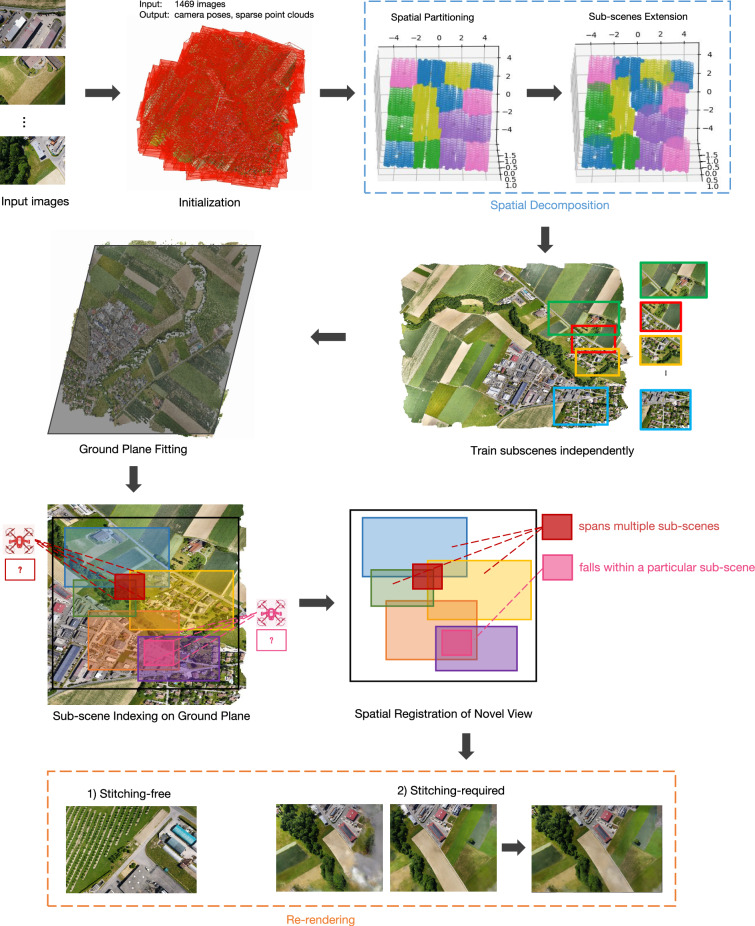
The BirdNeRF pipeline is initiated by the preprocessing phase, where input images are processed to obtain camera positions and sparse point clouds. Next, spatial decomposition is performed, categorizing cameras into clusters. For each cluster, associated images facilitate independent training, creating multiple sub-scenes. Finally, the projection-guided novel view re-rendering strategy synthesizes the final rendered images.

As depicted in Fig. 2, BirdNeRF encompasses two principal phases: (1) spatial decomposition, which involves dividing the scene into manageable, cluster-based segments, and (2) projection-guided novel view re-rendering, which reunites the independently processed segments to render the desired viewpoint.

### Background

BirdNeRF is fundamentally based on the original NeRF^10^ methodology and its subsequent advancement, Instant-NGP^13^. Here, we provide a synopsis of these foundational methods, while detailed expositions can be found in the respective source papers.

#### NeRF overview

NeRF is a transformative model that introduces a fresh paradigm to scene representation and view synthesis, enabling the creation of photorealistic 3D scenes from a set of 2D images. NeRF operates by associating each pixel in an image with a corresponding ray in 3D space, estimating color and density along these rays, and adjusting network parameters to minimize the difference between observed and predicted colors. Once trained, NeRF can be leveraged to synthesize novel views by projecting rays from new camera positions and integrating the color and density estimates to produce lifelike images from viewpoints not captured in the original dataset.

To elucidate the principle of Neural Radiance Fields (NeRF), we begin by considering a point with specific pixel coordinates located at the center of the camera. Starting from this point, a ray can be defined as:

1 $$\begin{aligned} \textbf{r}(t) = \textbf{o} + t \textbf{d}, \end{aligned}$$

where $\textbf{o}$ denotes the camera origin and $\textbf{d}$ is the normalized direction vector pointing towards the pixel on the image plane.

Points along this ray are parameterized by their spatial position $\textbf{x} = [x, y, z]$ and direction $\textbf{d} = [d_1, d_2, d_3]$. Each position and direction is encoded with a high-frequency positional encoding to facilitate the learning of intricate scene detail. Given a scalar input *x*, the positional encoding $\gamma (x)$ is defined as:

2 $$\begin{aligned} \gamma (x) = \left( \sin (2^0 \pi x), \cos (2^0 \pi x), \ldots , \sin (2^{L-1} \pi x), \cos (2^{L-1} \pi x) \right) , \end{aligned}$$

where *L* is the number of levels of positional encoding, NeRF sets $L = 10$ for spatial locations $\gamma (\textbf{x})$ and $L = 4$ for view directions $\gamma (\textbf{d})$.

Subsequently, this encoded positional and directional information is fed into the NeRF model, which is a Multi-Layer Perceptron (MLP), ultimately outputting the color $\textbf{c} = [r, g, b]$ and density $\sigma$ for each point.

To generate the color of a pixel, NeRF accumulates color and density predictions along the ray using volumetric rendering. The final color estimate $\hat{C}(\textbf{r})$ for a ray $\textbf{r}$ is given by the following discrete formulation:

3 $$\begin{aligned} \hat{C}(\textbf{r}) = \sum _{i=1}^N T_i \left( 1 - \exp (-\sigma _i \delta _i)\right) \textbf{c}_i, \quad \text {where} \quad T_i = \exp \left( - \sum _{j=1}^{i-1} \sigma _j \delta _j \right) , \end{aligned}$$

and $\delta _{i}$ denotes the distance between consecutive samples, i.e., between points $p_i$ and $p_{i+1}$ along the ray.

To optimize the model, a loss function $\mathscr {L}$ is computed by measuring the mean squared error between the rendered color and the ground truth value for each ray:

4 $$\begin{aligned} \mathscr {L} = \sum _{\textbf{r} \in \mathscr {R}} \Vert C(\textbf{r}) - \hat{C}(\textbf{r}) \Vert ^2, \end{aligned}$$

where $\mathscr {R}$ is the set of rays in each batch, $C(\textbf{r})$ is the ground-truth color, and $\hat{C}(\textbf{r})$ is the color predicted by the model.

Through this formulation, NeRF is able to construct an implicit representation of a 3D scene, enabling the synthesis of high-quality images from arbitrary camera viewpoints.

#### Instant-NGP

 Instant-NGP^13^ is a high-performance neural radiance field method developed by NVIDIA. It stands out by utilizing a multi-resolution hash encoding strategy, which dramatically accelerates training. The approach incorporates an advanced multilevel hash table to store trainable feature vectors, significantly reducing the memory footprint and overall model size. As a comprehensive framework, Instant-NGP enables seamless and efficient training as well as real-time rendering for detailed neural representations.

The key contribution of Instant-NGP is a novel multi-resolution hash encoding that maps input coordinates to a higher dimensional embedding space. This encoding employs a cascade of hash tables across multiple resolutions, with exponentially increasing resolution from coarse to fine levels. The hash tables help disambiguate hash collisions across resolution levels, providing a high-capacity input representation for neural networks.

##### Multi-resolution Hash Encoding

The input coordinate $\textbf{x}$ is encoded using *L* levels of hash tables, each with up to *T* entries of F-dimensional feature vectors. At level *l*, the encoded value is computed as:

5 $$\begin{aligned} \textrm{enc}(\textbf{x}, l) = \sum _{i=0}^{T-1} \phi (h(\textbf{x}, l), i, l) \cdot w(\textbf{x}, h(\textbf{x}, l), i, l) , \end{aligned}$$

where $h(\textbf{x}, l) = hash(\lfloor \textbf{x} \cdot N_l \rfloor ) \% T$ ( $N_l$ is the resolution at level *l* ), $w(\textbf{x}, h, i, l)$ is trilinear interpolation weights based on $(\textbf{x} \cdot N_l - \lfloor \textbf{x} \cdot N_l \rfloor )$, $\phi (i, j, l)$ retrieves the j-th feature vector at index i in the hash table of level l.

The final encoding is the concatenation of encodings from all levels:

6 $$\begin{aligned} \textrm{enc}(\textbf{x}; \theta ) = \left[ \textrm{enc}(\textbf{x}, 0); \textrm{enc}(\textbf{x}, 1); ... ; \textrm{enc}(\textbf{x}, L-1)\right] , \end{aligned}$$

where $\theta$ is trainable encoding parameters.

This encoded vector $\textbf{y}=enc(\textbf{x}; \theta )$ is passed through a small neural network $m(\textbf{y}; {\Phi })$ to produce the output. The hash table sizes T and number of levels L are hyperparameters that control quality vs performance tradeoff. During training, the feature vectors $\phi (i, j, l)$ in the hash tables are optimized using stochastic gradient descent along with the network weights ${\Phi }$.

Combined with multiresolution encoding, the use of compact neural networks leads to considerable reductions in both computational cost and memory requirements, compared to conventional large MLPs acting directly on input coordinates. Instant-NGP further benefits from an efficient GPU implementation based on fully fused CUDA kernels, supporting parallel encoding and network evaluation, mixed-precision computation, and cache-friendly per-level hash lookups.

### Initialization

The datasets used in our experiments consists of large-scale aerial survey images. From these input images, we utilize COLMAP^8^, a classical MVS software, to estimate camera poses and generate a sparse point cloud representing the scene. This initialization step is fundamental and indispensable not only for traditional multi-view stereo reconstruction, but also for our proposed BirdNeRF framework. The accuracy of camera pose estimation directly impacts the quality of subsequent reconstructions.

We set the camera model as a pinhole camera model and used normalized and unified camera intrinsic parameters for subsequent calculations. Following this process, we perform incremental reconstruction to obtain a sparse point cloud, along with pose estimates for all cameras in the dataset. These preliminary outputs, including the estimated camera poses and the reconstructed sparse point cloud, serve as the essential foundation for the subsequent spatial decomposition strategy. They are also critical for the effective implementation of the entire algorithm.

### Spatial decomposition

#### Spatial partitioning

 After the initialization step, we obtain the intrinsic and extrinsic camera parameters, as well as a sparse point cloud. We introduce, for the first time, the use of K-Means clustering^40^ to perform initial spatial partitioning based on the 3D positions of camera centers. Unlike traditional heuristic or grid-based methods, this data-driven approach adapts to the actual spatial distribution of camera viewpoints. This strategy provides a principled and efficient solution for decomposing large-scale scenes in 3D reconstruction tasks.

Our method partitions the dataset by applying the K-Means algorithm to the spatial distribution of camera positions. The optimal number of clusters, K, is determined by the available GPU memory during training. The data corresponding to each cluster will be used to train different sub-scene. The main steps are as follows:

1. Set the maximum number of cameras per cluster, denoted as $\text {MaxNum}$. The number of clusters is calculated as $K = \text {round}\left( \frac{N}{\text {MaxNum}}\right)$, where N is the total number of cameras and round($\cdot$) denotes rounding to the nearest integer.
2. Initialize K cluster centers by choosing K camera positions as the initial centroids $(\mathbf {O_1},\mathbf {O_2},...,\mathbf {O_K})$. Let the clustering be denoted as $(\mathbf {C_1},\mathbf {C_2},...,\mathbf {C_K})$ The objective is to minimize the total within-cluster squared error: $E = \sum _{i=1}^{K} \sum _{X \in C_i} \Vert X - O_i \Vert ^2_2$.
3. For each camera position $\mathbf {X_i}$, compute the distance to each $\mathbf {O_j}$ and assign $\mathbf {X_i}$ to the cluster with the closest center.
4. Update cluster centers. For each cluster $C_j$, update its centroid as $O_j = \frac{1}{|C_j|} \sum _{X \in C_j} X$.
5. Repeat steps 3 and 4 until the change in error *E* falls below a preset threshold or the maximum number of iterations is reached.

After the initial clustering, a set of camera clusters is obtained. The images and corresponding camera parameters within each cluster can be used to train a separate sub-scene block. However, the initial clustering does not guarantee sufficient camera overlap between clusters, meaning that overlap between sub-scenes cannot be ensured. As a result, when combining rendered outputs from multiple sub-scenes, image registration may fail, which can negatively impact the quality of the final rendered results. Therefore, further processing of the initial camera clusters is necessary to address these issues.

#### Sub-scenes extension

**Fig. 3:**
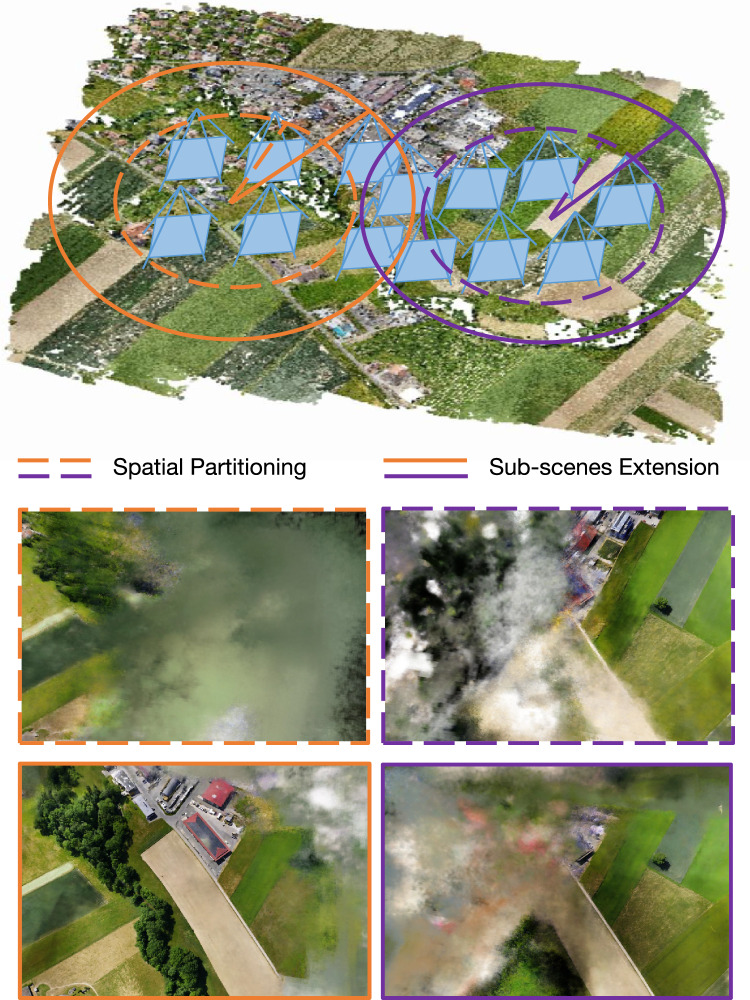
Sub-scenes extension. The strategic expansion of sub-scenes enhances scene overlap, thereby elevating the success rate of post-image registration in our proposed approach.

 We extend the sub-scenes to ensure a defined degree of overlap, enhancing the efficacy of image registration, as depicted in Fig. 3. Concretely, we introduce an expansion threshold denoted as $\sigma$, guiding the augmentation of each scene to guarantee a predetermined level of overlap within the partitioned sub-scenes. Specifically, after obtaining the initial camera clusters, we calculate the maximum intra-cluster radius for each cluster and multiply it by a predefined expansion threshold to determine the new radius for cluster expansion. Taking the current cluster center as the reference, we iteratively search all cameras within this new radius and add them to the corresponding cluster. It is worth noting that the proposed method imposes a maximum limit $\text {TopNum}$ on the number of images for each sub-scene to ensure smooth training within the allocated GPU memory resources. The camera parameters and corresponding images in each resulting camera cluster together constitute the training input for each sub-scene. The detailed steps are as follows:

1. For each initial camera cluster $\mathbf {C_i} (i=1,2,...,K)$, designate the cluster center as $\mathbf {O_i}$. Compute the distance between each camera in $\mathbf {C_i}$ and the center $\mathbf {O_i}$. Define the maximum such distance as the original cluster radius $r_i$, $r_i = \max _{X \in C_i} \Vert X - O_i \Vert$. The upper bound for cameras in the expanded cluster is set by $\text {TopNum}$.
2. Apply the expansion threshold $\sigma$ to obtain the new cluster radius $r'_i$, $r'_i = \sigma \times r_i$. Take $\mathbf {O_i}$ as the cluster center, and traverse all cameras in the dataset. For each camera, calculate its distance to $\mathbf {O_i}$. If the camera lies within the new radius $r'_i$, add it to the expanded cluster. Continue this process, adding cameras one by one to the cluster, ensuring the number of cameras remains within $\text {TopNum}$.
3. Repeat the above steps for all initial clusters. When the expansion for each cluster is complete, the spatial partitioning of the sub-scenes is finalized.

This expanded clustering method ensures that cameras located within the newly defined radius are explicitly added to the corresponding cluster, thereby improving the overlap between neighboring sub-scenes and enhancing the robustness of subsequent multi-block rendering and image alignment processes. While it is generally known that increased overlap between sub-scene clusters is beneficial for the quality of subsequent re-rendering, it is also critical to reserve a certain amount of GPU memory headroom to account for resource fluctuations and ensure training stability. In practice, we typically set the maximum number of cameras per cluster for initial K-Means partitioning (denoted as $\text {MaxNum}$) to 80% of the maximum number of training images the current GPU memory can reliably process in a single batch (denoted as $\text {TopNum}$). we introduce a default cluster expansion rate of 10% (i.e., an expansion threshold $\sigma =1.1$), explicitly allowing clusters to incorporate images from slightly broader spatial neighborhoods after the initial partitioning. This conservative parameter configuration is motivated by considerations of hardware safety, practical algorithm deployment, and the need to avoid GPU memory overflow. However, since this expansion could, in some edge cases, cause a cluster’s camera count to exceed the calculated MaxNum and risk surpassing memory limits, we enforce a hard upper bound by capping each cluster at TopNum cameras after expansion. This dual mechanism, which sets the initial clustering size to 80% of the hardware limit and then expands each cluster by 10% to improve overlap, both enhances re-rendering quality and reserves a 10% safety margin. As a result, the approach effectively balances memory utilization and hardware reliability.

This stage signifies the completion of camera division, denoting the subdivision of the scene into distinct sub-scenes. A comprehensive algorithmic depiction is presented in Algorithm 1.

**Algorithm 1:**
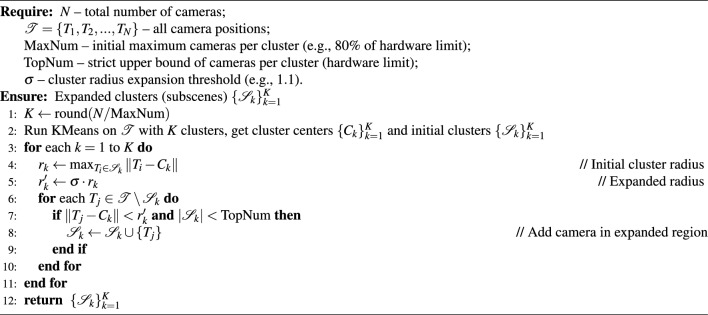
Spatial decomposition algorithm

### Individual training

After the spatial decomposition process, we obtain separate camera clusters where the camera parameters and corresponding images within each cluster serve as the training data for the respective sub-scenes. Independent training is conducted for each sub-scene using Instant-NGP as the base training model. Once training is complete, the resulting model parameters are stored offline on disk for future use in the re-rendering process. Notably, the models are saved as network parameters, which occupies significantly less disk space compared to alternative representations such as point clouds or grids. This efficient storage of the model parameters enables easier access and retrieval when needed.

### Projection-guided novel view re-rendering

**Fig. 4:**
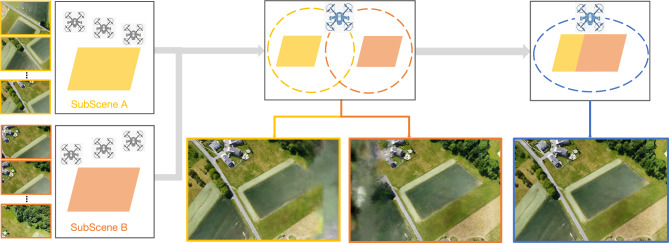
Projection-guided novel view re-rendering. Beginning with independently constructed input NeRFs, namely Sub-scene A and Sub-scene B, we perform image rendering from novel viewpoints. Then, employing a sequence of image stitching and fusion techniques, we achieve higher-quality re-rendering results.

To efficiently synthesize novel views from a collection of independently trained NeRF sub-scene models, we propose a projection-guided novel view re-rendering strategy. It automates the selection and fusion of relevant sub-scenes for any given query camera pose, as shown in Fig. 4. This pipline is the most conceptually innovative component of our framework.

Rather than relying on costly global scene optimization during neural model training, which is both memory- and time-intensive, we decouple the process and defer global consistency to the inference stage. Theoretically, this is achieved by explicitly leveraging the geometric constraints of projection: for any queried novel view, the projection relation between the camera and the ground plane is exploited to guide which sub-scenes will contribute, and how their renderings are spatially registered and fused. This allows us to train local sub-scene models independently but achieve consistent global rendering at inference by aligning and compositing their outputs via projective geometry. The fusion stage integrates multi-band blending and photometric normalization techniques, ensuring seamless transitions across sub-scene boundaries and robust rendering quality. This theoretically grounded separation of learning and composition dramatically improves efficiency and scalability while maintaining high visual fidelity.

#### Ground plane fitting

 Prior to advancing to the subsequent stages of the program, it is essential to robustly estimate the parameters of the ground plane, which serves as the geometric reference for all subsequent projection and fusion steps in our pipeline. We achieve this by applying the Least Squares^41^ method to the sparse point cloud obtained during the initialization phase. Specifically, Our approach utilizes all 3D points from the sparse point cloud denoted as $\{\textbf{P}_j=(x_j,y_j,z_j)\}^N_{j=1}$. Incorporating all sparse points into the fitting process significantly improves the robustness and global consistency of the estimation, effectively mitigating the influence of noise and outliers that might be present in subsets of the data. The ground plane is modeled by the equation $ax+by+cz+d=0$, where (*a*, *b*, *c*) denotes the normal vector of the plane and *d* is the offset term. The optimal plane parameters are obtained by solving the following constrained least squares problem: $\min _{a, b, c, d} \sum _{j=1}^N [a x_j + b y_j + c z_j + d]^2$ subject to the normalization constraint $a^2 + b^2 + c^2 = 1$. Fitting the plane to the entire sparse point set ensures that the estimated ground plane accurately reflects the global structure of the scene, providing a stable and reliable geometric foundation for indexing sub-scenes in the later projection-guided novel view re-rendering pipeline.

#### Sub-scene indexing on ground plane

 We employ a methodology that projects the four corner points of each image onto the ground plane, allowing us to precisely determine the spatial extent of the scene captured by each camera. This procedure is crucial for efficient region indexing and subsequent rendering within our system.

Camera models are mathematical abstractions describing image formation. We utilize the pinhole camera model, which maps 3D world points onto a 2D image plane via perspective projection. In our setting, the effect of lens distortion is negligible, so this model suffices for accurate scene projection.

The normalized direction vectors, pointing from the camera optical center through each image corner, are:

7 $$\begin{aligned} \textbf{d}_1^i = \begin{bmatrix} \frac{0 - c_x}{f_i} \\ \frac{0 - c_y}{f_i} \\ 1 \end{bmatrix}, \quad \textbf{d}_2^i = \begin{bmatrix} \frac{w - c_x}{f_i} \\ \frac{0 - c_y}{f_i} \\ 1 \end{bmatrix}, \quad \textbf{d}_3^i = \begin{bmatrix} \frac{w - c_x}{f_i} \\ \frac{h - c_y}{f_i} \\ 1 \end{bmatrix}, \quad \textbf{d}_4^i = \begin{bmatrix} \frac{0 - c_x}{f_i} \\ \frac{h - c_y}{f_i} \\ 1 \end{bmatrix} \end{aligned}$$

The optical center of the camera in the camera coordinate system is:

8 $$\begin{aligned} \textbf{o}^i = \begin{bmatrix} 0\\ 0\\ 0 \end{bmatrix} \end{aligned}$$

The transformation from the camera coordinate system to the world coordinate system, given rotation $R^i$ and translation $T^i$ , is applied to both the direction vectors and the optical center:

9 $$\begin{aligned} \textbf{r}_k^i(\lambda ) = R^i \big ( \lambda \cdot \textbf{d}_k^i \big ) + \textbf{T}^i, \quad \lambda > 0 \end{aligned}$$

Here, $\textbf{r}_k^i(\lambda )$ is the world coordinate of a point along the ray originating from the camera center and passing through the k-th image corner, parameterized by $\lambda$.

The world coordinate of the optical center is simply:

10 $$\begin{aligned} \textbf{O}^i = R^i \cdot \textbf{o}^i + \textbf{T}^i = \textbf{T}^i \end{aligned}$$

To project each ray onto the ground plane $ax+by+cz+d=0$, we solve for $\lambda ^*$ such that $\textbf{r}_k^i(\lambda ^*)$ satisfies the plane equation. The intersection points $\textbf{g}_k^i$ are:

11 $$\begin{aligned} \textbf{g}_k^i = \textbf{r}_k^i(\lambda ^*) \end{aligned}$$

where $\lambda ^*$ is obtained by substituting the ray into the plane equation and solving for $\lambda$:

12 $$\begin{aligned} a x(\lambda ) + b y(\lambda ) + c z(\lambda ) + d = 0 \end{aligned}$$

**Fig. 5:**
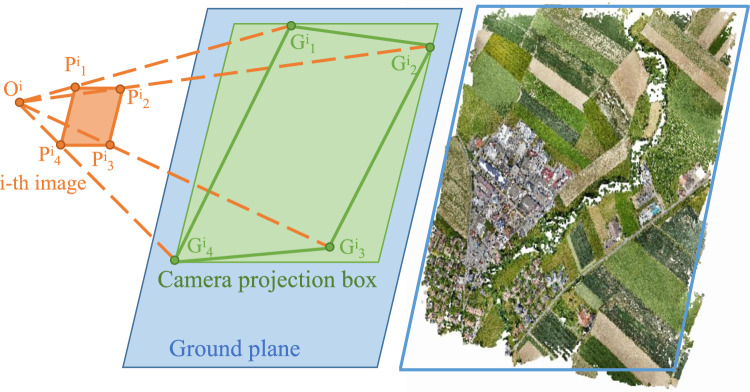
Ground plane fitting and pixel projection. By fitting the ground plane using sparse point cloud information of the scene, the four corner points of the image are then projected onto the ground plane.

This generates the four intersection points, as depicted in Fig. 5. The minimum rectangle that encompasses all such points is determined upon obtaining these intersection points. This rectangle effectively represents the extent of the scene captured by the $i-th$ camera, known as the camera projection box.

**Fig. 6:**
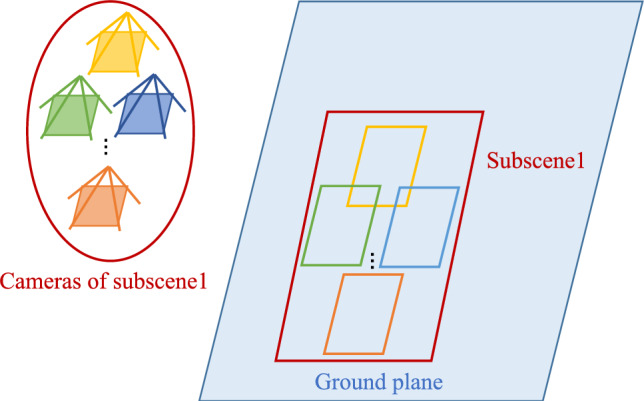
Sub-scene bounding Box. The projection boxes of all cameras within each divided sub-scene collectively form the projection box of the sub-scene range.

As detailed in previous Steps, we employ a spatial decomposition methodology to assign each camera to one or more sub-scenes. Each sub-scene includes a group of cameras, and its initial bounding box is defined as the union of the individual camera projection bounding boxes associated with the sub-scene. To further refine this region, we compute the minimum bounding rectangle that encompasses the entire scene range observed by all cameras in the sub-scene. This resulting rectangle is used as the scene bounding box for the sub-scene, which facilitates precise resource allocation and spatial indexing, as illustrated in Fig. 6.

**Fig. 7:**
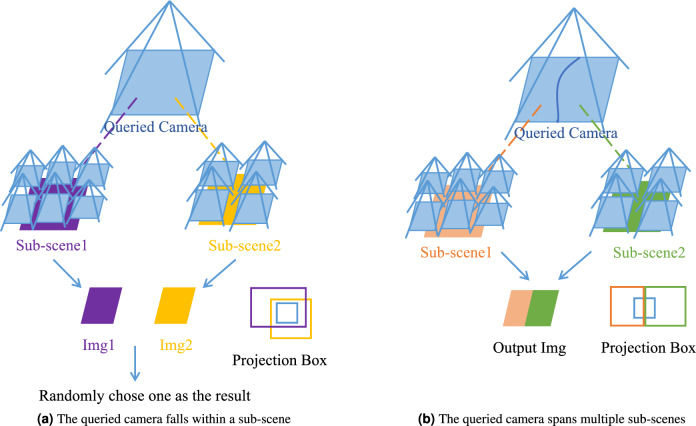
Two types of the queried camera position distributions. When the queried camera projection box is entirely contained within the bounding box of a specific sub-scene, the rendered image is directly output from that sub-scene. In cases where the queried camera is positioned at the boundary of multiple sub-scenes, registration, and fusion of outputs from these sub-scenes are necessary to obtain the final result.

#### Spatial registration of novel view

 In accordance with the methodology employed in the preceding section, we compute the projection box of the queried camera onto the ground plane. Subsequently, we systematically iterate through all the sub-scene bounding boxes to identify those intersecting with the queried camera’s projection box. These identified sub-scene bounding boxes are preserved as rendering schemes, guiding the subsequent rendering process for the queried image. This rendering process is executed by leveraging the trained models associated with each respective sub-scene. This targeted selection ensures that only the necessary sub-scenes are considered in downstream processing, enhancing both computational efficiency and output quality.

#### Re-rendering

 Once we obtain the bounding boxes for each independently trained sub-scene, we can automatically generate a rendering strategy for the queried camera that needs to be rendered. Generally speaking, there are two situations when querying scenes (Fig. 7):

##### Stitching-free

When the projection box of the queried camera lies entirely within a single sub-scene’s bounding box (see Fig. 7a ), re-rendering is straightforward. In this case, the corresponding sub-scene model alone is sufficient to generate the complete novel view. The output can be directly obtained from the rendered results of the selected sub-scene, without any need for cross-scene fusion or blending operations.

##### Stitching-required

**Fig. 8:**
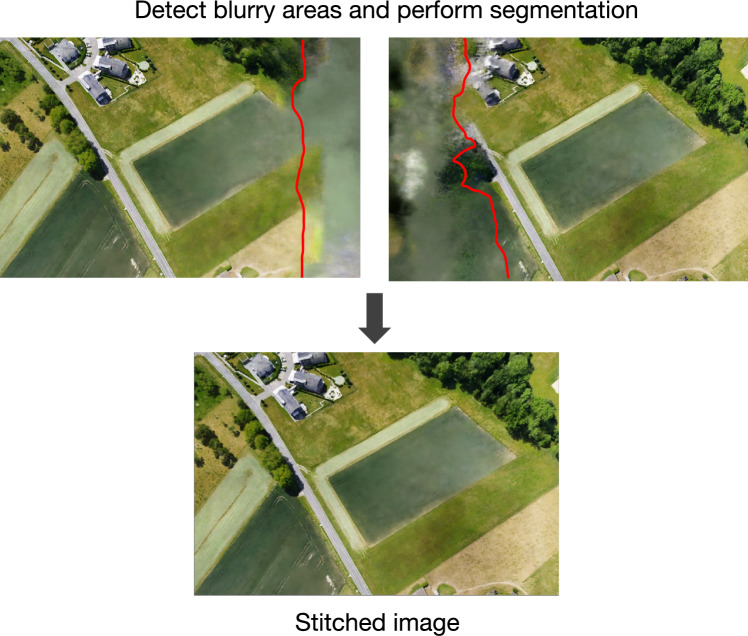
Blur area detection and image stitching. Blur removal prior to image stitching yields optimal results.

When the projection box of the queried camera overlaps with multiple sub-scene bounding boxes (see Fig. 7b ), the camera view extends across several sub-scenes. In this situation, we render the relevant portions using each corresponding sub-scene model separately. The outputs are then registered and composited to form the final image. To address inconsistencies or potential artifacts at the seams, we first detect and suppress blurred areas from each result^42^, as delineated in Fig. 8, then employ an advanced image stitching pipeline^43,44^ that incorporates gain compensation, simple blending, and multi-band blending to produce a seamless and coherent output. Theoretically, this multi-level fusion approach, grounded in both projective geometry and frequency-domain image processing,ensures that large-scale scene rendering is both seamless and scale-adaptive. By systematically resolving domain inconsistencies at sub-scene boundaries, our method advances the rendering fidelity and robustness for novel views in complex, expansive environments.

### Discussion

This paper provides a comprehensive solution for reconstructing large-scale 3D scenes from a massive number of aerial images, achieving the fastest large-scale 3D reconstruction to date while ensuring rendering quality. There are also several noteworthy points and discussions:

- **Novelty over Instant-NGP?** This method is a kind of general strategy-based approach. The selection of the benchmark model can theoretically be any effective deep learning-based 3D reconstruction model, primarily used for training in sub-scenes. Furthermore, this method is an effective solution for large-scale 3D reconstruction based on the principles of divide-and-conquer.
- **Fastest Large-Scale Modeling Methods to Date?** Based on the need for fast large-scale reconstruction modeling, we chose the currently fastest neural radiance field method Instant-NGP as the baseline model for training the various sub-scene models. From the perspective of the overall time required to complete the task, our method can meet the near-real-time requirements and provides the fastest large-scale 3D scene reconstruction method to date.

## Experiments

### Implementation

We run our method on a 12th Gen Intel(R) Core(TM) i9-12900KF with 32GB RAM. All experiments in this paper are conducted on a single NVIDIA GeForce RTX 3090 GPU(24GB). We set the initial maximum number of cameras per cluster to 90 to determine the initial clustering clusters (K value). Additionally, we set the maximum number of cameras in each partitioned sub-scene to 113 to ensure our method runs without GPU memory overflow. This value can be adjusted according to the GPU specifications of the experimental environment. We set the expansion threshold $\sigma$ to 1.1 when scaling the maximum intra-cluster distance for camera cluster augmentation. For the model training in each sub-scene, we set the number of training iterations to $5 \times 10^3$, while keeping other parameters at their default values.

### Datasets

Our experiments use aerial datasets from 8 distinct geographical regions, each differing in size and characteristics. The datasets are sourced from both publicly available repositories and those captured with the DJI Mavic Air 2 drone in Hexi University Town, Changsha. Together, these datasets encompass urban, suburban, industrial, agricultural, and university campus environments, offering diverse scenes for thorough evaluation.

We select three real photogrammetry datasets from Pix4D’s example projects^45^:

- **Urban area(UA).** UA represents a city dataset covering 0.0214 $km^2$, featuring 100 images with a resolution of $6000 \times 4000$ pixels.
- **Suburban area(SA).** SA comprises 188 images at a resolution of $5472 \times 3648$ pixels, covering an area of 0.041 $km^2$.
- **Industrial zone and agriculture area(IZAA).** IZAA dataset consist of 1469 images at a resolution of $6000 \times 4000$ pixels, encompassing an extensive area of 1.154 $km^2$. This dataset includes diverse regions such as an industrial zone, suburban residential areas, and surrounding agricultural landscapes.

Utilizing the DJI Mavic Air 2 drone, we conduct aerial surveys capturing five distinct regions within Hexi University Town, located in Changsha, Hunan Province. These surveys produce five datasets, ranging in size from hundreds to thousands of images. Notably, the flight trajectories of our drone missions introduce additional complexities compared to the publicly available datasets mentioned earlier.

- **CSU1.** The CSU1 dataset contains 408 images with a resolution of $4000 \times 3000$ pixels, covering an area of approximately 0.2 $km^2$, including the sports stadium and gymnasium areas of the new campus at Central South University.
- **CSU2.** The CSU2 dataset comprises 713 images with a resolution of $4000 \times 3000$ pixels, covering approximately 1/3 of the southwest area of the new campus at Central South University. The total area covered is approximately 0.2 $km^2$.
- **HNU.** The HNU dataset consists of 391 images with a resolution of $4000 \times 3000$ pixels, covering an area of approximately 0.1 $km^2$ around the Tianma Student Dormitory at Hunan University.
- **CSUS.** The CSUS dataset consists of 777 captured photos with a resolution of $4000 \times 3000$ pixels, covering an on-site area of approximately 0.7 $km^2$ in the Southern Campus of Central South University.
- **CSUHU.** The CSUHU dataset comprises 1706 photos covering an approximate area of 1 $km^2$. The main shooting locations include parts of the New Campus of Central South University and portions of the Houhu International Art Park buildings. The images in the dataset have a resolution of $4000 \times 3000$ pixels.

### Ablation study

To systematically evaluate the design choices of our method, we conduct comprehensive ablation studies on the CSUHU dataset, which consists of 1706 images. we analyze how the overlap ratio, number of sub-scenes, and training iterations affect performance in large-scale reconstruction on a single 24GB GPU. Unless otherwise noted, default settings from our main experiments were adopted.

#### Impact of Overlap Ratio and Number of Sub-scenes

 As detailed in Section Spatial decomposition, the number of sub-scenes is determined by the MaxNum parameter, defined as a percentage of the strict upper bound of cameras per cluster (TopNum). The overlap ratio is reflected in the expansion threshold $\sigma = 1 + \text {overlap ratio}$, and both the overlap ratio and the predefined MaxNum are constrained by GPU memory capacity. We conduct ablation experiments for MaxNum values set to 70%, 80%, and 90% of TopNum (MaxNum=79, 90, 102), with the corresponding number of sub-scenes calculated according to $K = \text {round}(N / \text {MaxNum})$.

As shown in Table 1, when $\text {MaxNum}= 80\% \times \text {TopNum} = 90$ with a 10% overlap ratio, the model has the best PSNR and SSIM scores. When MaxNum is set to $\text {MaxNum} = 70\% \times \text {TopNum} = 79$,the number of sub-scenes becomes large, resulting in each sub-scene containing insufficient training images. This leads to suboptimal reconstruction quality across all tested overlap ratios. For $\text {MaxNum} = 80\% \times \text {TopNum} = 90$, with a 20% overlap ratio, as well as $\text {MaxNum} = 90\% \times \text {TopNum} = 102$ with a 10% overlap, we observe sporadic failures in a small subset of sub-scenes during training, which degrades the overall rendering results. In other settings (e.g., higher MaxNum combined with larger overlaps), the GPU runs out of memory and model training cannot proceed.

**Table 1:** Ablation Study of Overlap Ratio and Number of Sub-scenes on Dataset CSUHU.

	MaxNum	Number of Sub-scenes	Overlap Ratio	PSNR $\uparrow$	SSIM $\uparrow$
CSUHU	79	22	10%	17.376	0.450
			20%	20.293	0.481
			30%	21.109	0.558
	90	19	10%	**21.580**	**0.564**
			20%	20.825	0.540
			30%	/	/
	102	17	10%	18.642	0.479
			20%	/	/
			30%	/	/

#### Impact of Training Iterations

 To assess the effect of training iterations, we vary the number of iterations from $5 \times 10^3$ to $3 \times 10^4$ and report results in Table 2. The findings indicate that model performance, in terms of both PSNR and SSIM, begins to saturate beyond $5 \times 10^3$ iterations. While further increases in training iterations yield marginal gains in reconstruction quality, these come at a substantial cost in training time. In particular, training each sub-scene for $5 \times 10^3$ iterations already achieves a favorable balance between computational efficiency and visual fidelity.

**Table 2:** The PSNR and SSIM scores for different training iterations of our method BirdNeRF on CSUHU.

	Training iterations	Training time(h) $\downarrow$	PSNR $\uparrow$	SSIM $\uparrow$
CSUHU	$5 \times 10^3$	0.341	21.504	0.659
	$1 \times 10^4$	0.671	21.763	0.670
	$3 \times 10^4$	1.927	22.132	0.687

### State-of-the-art comparison

#### Comparison methods

In our experiments, the proposed method BirdNeRF is compared to three existing city-level reconstruction solutions that can be modeled with a single GPU:

- **Metashape **^33^: Agisoft Metashape is a commercial software designed to process digital images using photogrammetry methods. We conducted our control experiments using Agisoft Metashape Pro 1.6.5.
- **Mega-NeRF **^5^: Mega-NeRF is a scene segmentation method, but it performs segmentation at the pixel level.
- **Instant-NGP **^13^: Instant-NGP is currently the fastest neural radiance field training method, and it serves as a benchmark for our approach BirdNeRF.

Please note that in the current experimental setup, Instant-NGP is only capable of processing a maximum of 110 images at a resolution of $6000 \times 4000$ for scene modeling, resulting in a limited reconstruction scope as shown in Table 3. Therefore, we evaluated and compared this method only on the small UA dataset, which contains only 100 images, excluding it from other comparisons.

**Fig. 9:**
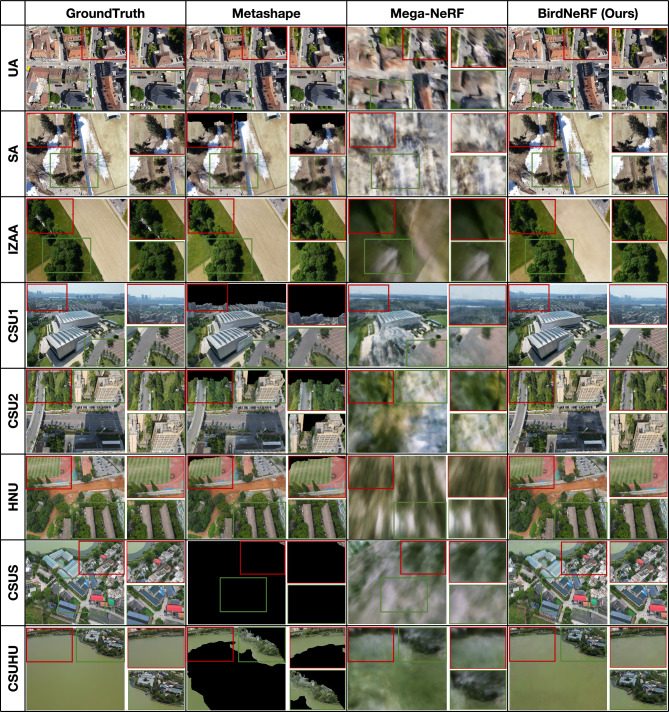
Comparison of rendered results. Qualitative comparison of image quality among Metashape, Mega-NeRF, and BirdNeRF indicates the clear superiority of BirdNeRF.

#### Evaluation metrics

We quantify the performance of our method using two established quantitative metrics: peak signal-to-noise ratio (PSNR) and structural similarity (SSIM). These metrics accurately assess the quality and similarity between the rendered images and their corresponding ground truth images. Additionally, we assess the efficiency of different methods by comparing the training time required after aligning the cameras in a consistent training environment. This dual evaluation framework provides a comprehensive analysis, addressing our proposed method’s quality and efficiency dimensions.

**Fig. 10:**
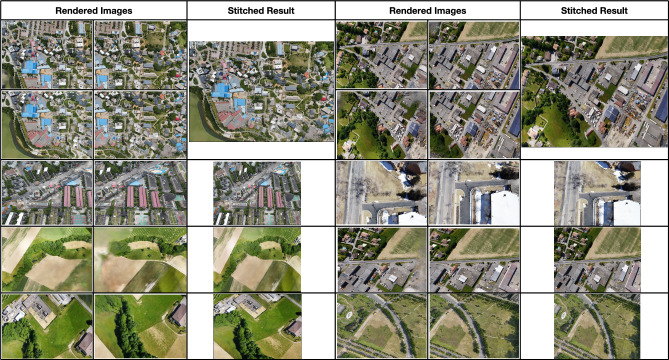
Stitching-required examples. The images generated from individual sub-scenes are subsequently concatenated to produce the final result through a stitching process.

#### Qualitative results

Figure 9 presents a comprehensive visual comparison of the final rendered results from Metashape^33^, Mega-NeRF^5^, and our proposed method BirdNeRF across eight diverse aerial datasets. This figure provides an effective demonstration of each method’s performance on large-scale scene modeling. Metashape frequently produces undesirable holes or incomplete regions (missing areas), and visually unrealistic artifacts often appear when image coverage is sparse or in challenging viewpoints. Mega-NeRF, while representing a great advance in neural radiance field modeling for large scenes, tends to generate blurred results and loses crucial structural details in several datasets. In contrast, BirdNeRF consistently achieves highly complete and visually coherent reconstructions, closely matching the ground truth in both structure and overall appearance. Additionally, to rigorously validate the effectiveness and generalizability of our approach, we intentionally employ randomly generated camera poses during the validation phase. Furthermore, we present examples of stitch-required in Fig. 10, depicting cases where the queried camera is positioned at the intersections of multiple sub-scenes.

#### Quantitative analysis

We conduct a quantitative analysis of the rendered results and training speed across different methods. Our findings reveal that our method excels in rapidly attaining high-quality results in large-scale 3D reconstruction, even under constraints of limited GPU memory resources.

##### PSNR and SSIM scores

 Table 3 depicts the PSNR and SSIM scores, offering a comparative analysis of the rendered results from all evaluated methods in this study against the ground truth images. Our method consistently achieves the highest PSNR scores across all datasets, securing SSIM superiority on nearly half of them, demonstrating greater stability in both metrics. Metashape displays a broader range of PSNR fluctuations. In contrast, our method maintains a consistently narrow range of fluctuations in scores, indicating its robustness and ability to deliver consistent and reliable results.

**Table 3:** PSNR and SSIM scores of rendered results from Metashape^33^, Mega-NeRF^5^,Instant-NGP^13^ and BirdNeRF.

Dataset	Method	PSNR $\uparrow$			SSIM $\uparrow$		
		Min	Max	Average	Min	Max	Average
UA	Metashape	13.619	24.713	19.891	0.890	0.963	**0.930**
	Mega-NeRF	9.860	13.106	11.382	0.244	0.355	0.282
	Instant-NGP	19.787	21.35	20.154	0.514	0.617	0.562
	BirdNeRF	19.603	20.768	**20.167**	0.563	0.6560	0.595
SA	Metashape	5.135	34.819	18.517	0.686	0.995	**0.935**
	Mega-NeRF	7.105	12.514	9.808	0.146	0.469	0.296
	Instant-NGP	$\backslash$	$\backslash$	$\backslash$	$\backslash$	$\backslash$	$\backslash$
	BirdNeRF	16.508	23.120	**20.002**	0.448	0.688	0.555
IZAA	Metashape	6.353	35.99	21.523	0.654	0.994	**0.887**
	Mega-NeRF	8.757	19.116	12.845	0.124	0.548	0.276
	Instant-NGP	$\backslash$	$\backslash$	$\backslash$	$\backslash$	$\backslash$	$\backslash$
	BirdNeRF	17.223	28.847	**21.829**	0.399	0.775	0.544
CSU1	Metashape	2.492	30.568	9.787	0.427	0.987	0.686
	Mega-NeRF	16.813	23.428	20.101	0.401	0.696	0.540
	Instant-NGP	$\backslash$	$\backslash$	$\backslash$	$\backslash$	$\backslash$	$\backslash$
	BirdNeRF	18.226	26.255	**22.579**	0.602	0.772	**0.689**
CSU2	Metashape	5.974	31.373	19.167	0.725	0.985	**0.911**
	Mega-NeRF	8.887	15.577	12.308	0.125	0.395	0.257
	Instant-NGP	$\backslash$	$\backslash$	$\backslash$	$\backslash$	$\backslash$	$\backslash$
	BirdNeRF	11.013	25.290	**19.868**	0.286	0.701	0.539
HNU	Metashape	5.806	28.251	21.127	0.723	0.988	**0.929**
	Mega-NeRF	10.236	13.852	11.937	0.188	0.319	0.252
	Instant-NGP	$\backslash$	$\backslash$	$\backslash$	$\backslash$	$\backslash$	$\backslash$
	BirdNeRF	17.664	22.954	**21.158**	0.427	0.618	0.552
CSUS	Metashape	0.000	39.632	12.885	0.000	0.998	0.541
	Mega-NeRF	10.102	13.325	12.023	0.227	0.423	0.314
	Instant-NGP	$\backslash$	$\backslash$	$\backslash$	$\backslash$	$\backslash$	$\backslash$
	BirdNeRF	19.287	23.907	**21.580**	0.448	0.659	**0.564**
CSUHU	Metashape	0.957	38.553	8.776	0.190	0.996	0.658
	Mega-NeRF	10.748	20.995	14.135	0.191	0.921	0.468
	Instant-NGP	$\backslash$	$\backslash$	$\backslash$	$\backslash$	$\backslash$	$\backslash$
	BirdNeRF	17.459	26.787	**21.505**	0.442	0.889	**0.659**

##### Runtime comparison

 Instant-NGP demonstrates rapid convergence after $3 \times 10^3$ training iterations, whereas Mega-NeRF, an enhanced version of the initial NeRF, requires approximately $1 \times 10^5$ iterations to reach convergence. To ensure sufficient training for both Mega-NeRF and our method BirdNeRF, we set the training iterations for our method to $5 \times 10^3$ and for Mega-NeRF to $1 \times 10^5$ iterations. In Mega-NeRF’s clustering mask partitioning stage, we set the grid dimension to 2x4, dividing each scene into 8 sub-scenes. Utilizing a single NVIDIA GeForce RTX 3090 GPU (24GB), the training environment allows for parallel training of two sub-scenes in Mega-NeRF. Additionally, the time Metashape^33^ consumes from sparse point cloud to mesh generation is considered the training time.

**Table 4:** A comparison of training time for various methods.

Method	Training Time(h) $\downarrow$		
	Metashape	Mega-NeRF	BirdNeRF
UA	0.041	17.109	0.014
SA	0.253	17.413	0.029
IZAA	3.047	16.863	0.300
CSU1	0.445	17.238	0.076
CSU2	1.365	17.067	0.125
HNU	0.842	18.178	0.059
CSUS	2.304	30.748	0.154
CSUHU	4.281	27.847	0.341

**Fig. 11:**
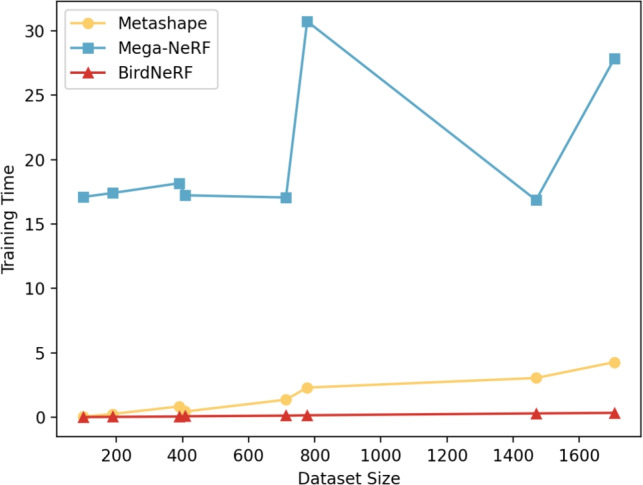
The trend of training time across eight datasets. BirdNeRF exhibits a slow increase in training time as the dataset size grows, with a more pronounced advantage observed on larger datasets.

Table 4 provides a comprehensive comparison of training times for various methods, highlighting the significant speed advantage of our approach. Figure 11 visually represents this result, clearly illustrating the overall lengthy training times of Mega-NeRF, based on the naive NeRF implementation. Metashape’s training time escalates quickly with the dataset size, whereas our method exhibits superior adaptability to large datasets with a slow increase in training time as the dataset grows.

The training time of our method remains independent of image resolution but is contingent on the number of training iterations. To showcase the effectiveness of our method, we compared training speed and rendered results on all datasets at different iteration counts. Table 5 presents the PSNR and SSIM scores for varying training iterations, revealing negligible differences in modeling performance. This flexibility allows us to adjust training iterations based on our time requirements.

**Table 5:** The PSNR and SSIM scores for different training iterations of our method BirdNeRF.

	Training iterations	Training time(h) $\downarrow$	PSNR $\uparrow$	SSIM $\uparrow$
UA	$5 \times 10^3$	0.014	20.167	0.595
	$1 \times 10^4$	0.029	20.432	0.610
	$3 \times 10^4$	0.093	20.830	0.636
SA	$5 \times 10^3$	0.029	20.002	0.555
	$1 \times 10^4$	0.063	20.236	0.568
	$3 \times 10^4$	11.667	20.725	0.590
IZAA	$5 \times 10^3$	0.300	21.829	0.544
	$1 \times 10^4$	0.599	22.231	0.554
	$3 \times 10^4$	1.730	22.725	0.569
CSU1	$5 \times 10^3$	0.076	22.579	0.690
	$1 \times 10^4$	0.149	23.306	0.713
	$3 \times 10^4$	0.461	23.766	0.738
CSU2	$5 \times 10^3$	0.125	19.868	0.539
	$1 \times 10^4$	0.253	20.107	0.557
	$3 \times 10^4$	0.753	20.329	0.582
HNU	$5 \times 10^3$	0.059	21.158	0.552
	$1 \times 10^4$	0.119	21.745	0.587
	$3 \times 10^4$	0.361	22.451	0.633
CSUS	$5 \times 10^3$	0.154	21.580	0.564
	$1 \times 10^4$	0.310	21.987	0.582
	$3 \times 10^4$	0.890	22.832	0.627
CSUHU	$5 \times 10^3$	0.341	21.504	0.659
	$1 \times 10^4$	0.671	21.763	0.670
	$3 \times 10^4$	1.927	22.132	0.687

## Conclusions

This paper introduces BirdNeRF, a fast neural reconstruction method designed for processing a large number of aerial images. It stands out as the fastest large-scale reconstruction method to date, emphasizing efficient memory resource utilization and high rendering quality. The spatial decomposition strategy, grounded in camera distribution clustering rather than naive geometric partitioning, enables BirdNeRF to form compact and data-aligned sub-scenes, enhancing scalability and system efficiency. In addition, the projection-guided novel view Re-rendering strategy is a core conceptual innovation. Instead of global optimization during training, it relies on geometric constraints and the projective relationship at inference time to guide fusion. This strategy guarantees accurate indexing of sub-scene bounding boxes and precise querying of related sub-models, ensuring high-quality rendering for diverse camera poses while significantly reducing computational cost. Evaluation results indicate substantial advancements over classical photogrammetric software and the state-of-the-art large-scale NeRF solutions. BirdNeRF achieves a speed increase of more than ten times on a single GPU while maintaining commendable rendering quality. BirdNeRF offers practical solutions for critical challenges, significantly enhancing the speed, scalability, and visual realism of large-scale aerial scene reconstruction. These improvements are especially valuable for real-time and time-critical applications, such as disaster response.

While BirdNeRF represents a substantial step forward, several avenues remain for future exploration. Currently, the system achieves near real-time performance but is still best suited for offline use. Achieving true real-time processing will require further algorithmic refinement, deeper optimization of low-level implementation, and more complete exploitation of GPU parallelism. In addition, BirdNeRF presently relies on traditional vision-based methods such as COLMAP for initial camera pose estimation. Future research will focus on integrating advanced deep learning-based pose estimation techniques and exploring tighter fusion between neural scene representation and pose recovery, both to streamline the workflow and to enhance robustness. Finally, continued development of lighter and more efficient neural architectures, as well as new activation and optimization strategies, promises to further reduce computation and memory requirements, empowering BirdNeRF to operate effectively even in more challenging and dynamic real-world environments.

## Data Availability

The datasets generated and/or analysed during the current study are available in the Github repository, https://github.com/Kikihqq/BirdNeRF-Dataset.
